# On the phonon dissipation contribution to nanoscale friction by direct contact

**DOI:** 10.1038/s41598-017-03046-8

**Published:** 2017-06-12

**Authors:** S. R. Sales de Mello, M. E. H. Maia da Costa, C. M. Menezes, C. D. Boeira, F. L. Freire Jr, F. Alvarez, C. A. Figueroa

**Affiliations:** 1 Centro de Ciências Exatas e da Tecnologia, Universidade de Caxias do Sul, Caxias do Sul-RS, 95070-560 Brazil; 2 Departamento de Física, Pontifícia Universidade Católica do Rio de Janeiro, Rio de Janeiro - RJ, 22453-900 Brazil; 3 Instituto de Física “Gleb Wataghin”, Universidade Estadual de Campinas, Campinas-SP, 13081-970 Brazil

## Abstract

The friction phenomenon is a ubiquitous manifestation of nature. Models considering phononic, electronic, magnetic, and electrostatic interactions are invoked to explain the fundamental forces involved in the friction phenomenon. In order to establish the incidence of the phonon prompting at the nanoscale friction by direct contact, we study a diamond spherical dome sliding on carbon thin films containing different amount of deuterium and hydrogen. The friction coefficient decreases by substituting hydrogen by deuterium atoms. This result is consistent with an energy dissipation vibration local mechanism from a disordered distribution of bond terminators.

## Introduction

The understanding of the physical causes and how controlling friction properties is a cutting edge challenge in order to save energy, diminishing wear, increasing the lifetime and sustainability of mechanical devices, and improving performance^1, 2^. From Leonardo da Vinci’s and Guillaume Amontons’s ancient experiments up to the present time, the friction effects continue demanding enforces to explain the observed phenomenon^3, 4^. Indeed, the non-conservative forces acting in the physical interaction between two surfaces in relative motion is not explained by a unique and fundamental physical mechanism. This is in part due to the complexity of the dissipative forces involved in the phenomenon, strongly depending on the length scales of the sliding parts^5–7^. Furthermore, one of the most challenging fields in tribology concerns with the connection between the engineering (macro) phenomenological models and physical fundamental (atomic and nanoscale) laws.

By the lack of better tools, molecular dynamics calculations are applied to inspect the macroscopic phenomenological *three-term kinetic friction model* (or part of it) to phenomena occurring at the nanoscale size^5, 6^. This model assumes the combination of three effects, namely, the adhesion force in the presence of a lubricant at zero normal load (Derjaguin offset), the coefficient of friction (da Vinci-Amontons-Coulomb law) and the effective shear stress (Bowden-Tabor law)^6^. Although this approach brings valuable information for practical applications, several basic answers remain pending as, for example, the physical nature of the Derjaguin offset, the physical understanding of the origin of the friction coefficient, and the influence of the shear stress on the phenomenom. Therefore, an attempt to improve the understanding of the *three-term kinetic friction model* by using fundamental physical properties of the matter could help to unify several mechanisms prompting the friction at the nanoscale size that seem disconnected at the present.

From a statistical thermodynamically point of view, the friction phenomenon is one of the physical manifestations of the *fluctuation-dissipation theorem* (FDT)^8^. This important theorem explains the transition from the microscopic reversibility physical process to a macro irreversibly phenomena involving energy dissipation, i.e., entropy increasing^9^. Recently, the FDT was invoked to explore the electrostatic coupling between induced dipoles of two atoms^10^. Moreover, different mechanisms dealing with phonons, electronic band transitions, magnetic and electrostatic interactions were proposed to explain nanotribological effects. Whatever the origin of the friction fundamental mechanism involved in the phenomenon, any study must start taking into account the energy exchange (fluctuation) and its subsequent dissipation between the sliding surfaces.

Several works have explored the physical nature of energy exchange, coupling interaction, and dissipation mechanisms affecting the phenomenological friction coefficient and shear stress between sliding surfaces^8^. For instance, the superconducting transition drops abruptly the friction force of niobium thin films and solid nitrogen along a lead surface^11, 12^. The abrupt modification of the friction coefficient is attributed to fundamental friction mechanisms related to electronic and phononic effects instead of magnetic effects, although spin friction was also observed^13^. Other example is a system composed by polymeric materials where electrostatic forces are invoked to explain the observed friction coefficient^14^.

According to the phononic friction mechanisms of energy dissipation, the friction coefficient stems from mechanical energy transfer (momentum) and posterior energy dissipation through phonon excitation and damping^15–18^. Regarding with friction dissipative forces involving isotopic effects, it is remarkable the pioneering studied by Cannara *et al*. using atomic force microscopy (AFM)^19^. These researchers studied the effect on friction due to chemisorbed hydrogen and deuterium atoms terminating the outermost monolayer of amorphous carbon thin film. The Cannara *et al*. work raised two important criticisms by Mo *et al*.^20^. First, the study did not take into account the influence of the hydrogen and deuterium atomic surface coverage. Second, the molecular dynamic simulation by Mo *et al*. shows a friction force independent of the adsorbate mass atoms^20^.

In the attempt to contribute to this debate, one of the goals of this work is to investigate the isotopic role on the dissipative forces acting at the nanoscale. In order to overcome the problem of the surface coverage ratio effect on the friction phenomenon, samples containing *hydrogen and/or deuterium in material bulk* were prepared^20, 21^.

In this paper, we discuss the issue of friction between a diamond spherical dome sliding on amorphous carbon thin films containing different amounts of deuterium and/or hydrogen that modifies the phonon-only distribution. Being more specific, the experimental results take into account the *physical contact* under pressure arising between two sliding surfaces in relative motion, i.e., the effect of the phonon distribution of different isotopic systems taking in account the physical intimate contact of the sliding surfaces at an indentation depth of ~75 nm.

## Results and Discussion

### Isotopic systems for phonon-only contribution

**Figure 1:**
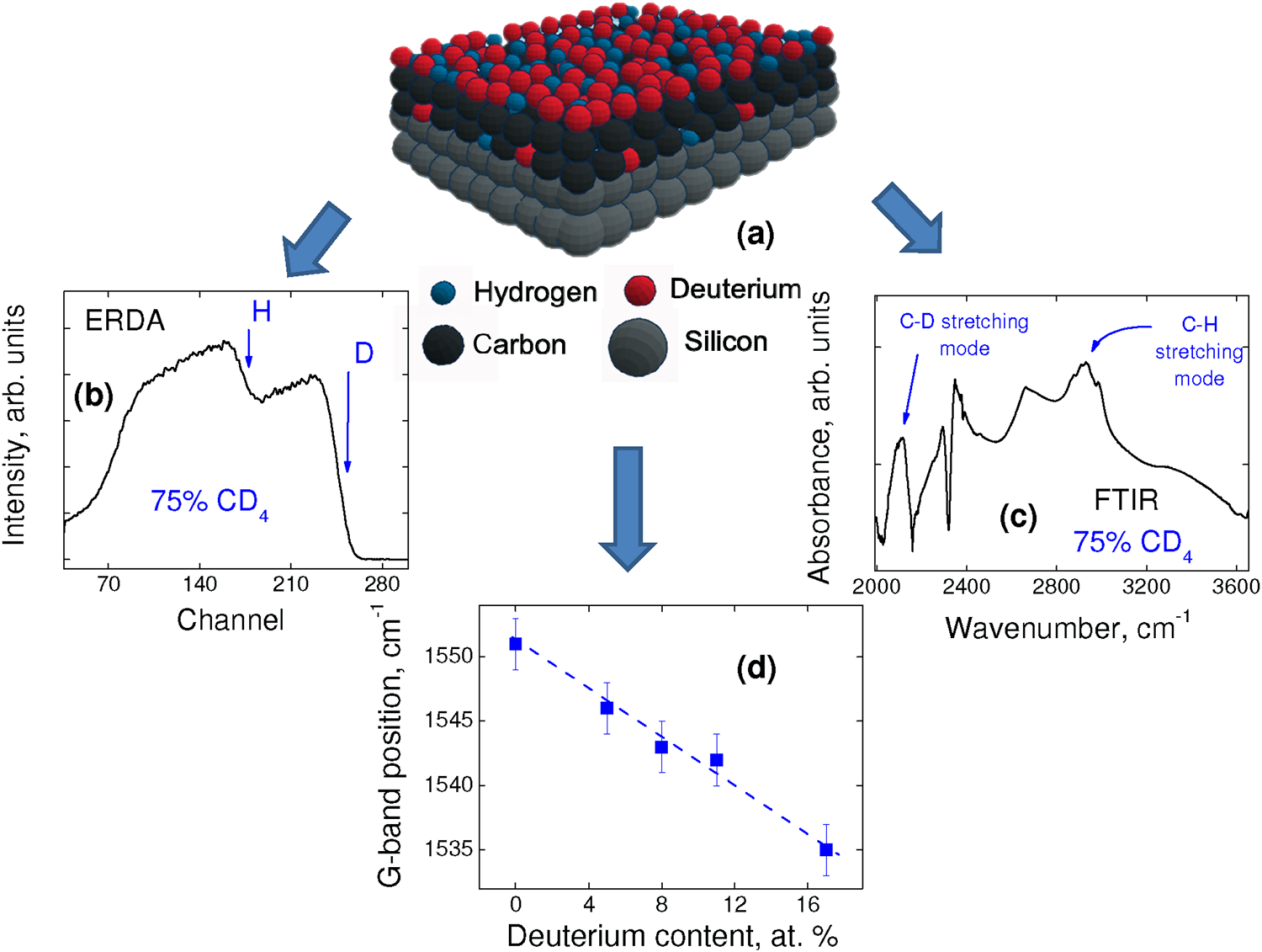
(**a**) Schematic of a typical a-C:D/H thin film deposited on a silicon substrate. (**b**) ERDA and (**c**) FTIR spectra obtained from the a-C:D/H thin film deposited with 75% of CD_4_ in the gas mixture. (**d**) G-band position from Raman spectroscopy of the a-C:D/H thin films as a function of the deuterium content.

Figure 1a schematically shows a model of the studied a-C:D/H thin films (isotopic systems). Figures 1b and c show the elastic recoil detection analysis (ERDA) and infrared (FTIR) spectra, respectively, for the sample deposited from a gas mixture of 25% of CH_4_ and 75% of CD_4_. Figure 1d shows the G-band position as a function of deuterium content obtained from Raman spectroscopy. The ERDA and Raman results for all the studied samples are similar (not shown).

As shown by the ERDA measurements, the deuterium signal rises before the hydrogen ones due to its higher atomic mass (see Fig. 1b)^22^. The signal width is proportional to the a-C:D/H thin film thickness and the signal shape (the slope after the maximum) is consistent with a scattering mechanism leading to conclude that both concentration profiles are constant throughout the film (see Fig. 1b). The Fourier transform Infrared - FTIR spectrum clearly shows the stretching modes associated with both C-D (~2120 cm^−1^) and C-H (~2940 cm^−1^) vibrations modes (Fig. 1b)^23^.

The Raman spectra for all the studied samples (not shown) allow identifying, indirectly, the presence of increasing deuterium in the material structure. The G-band corresponds to the bond stretching of all pairs of sp^2^ atoms in both rings and chains^24^. In fact, as expected, the G-band shifts to lower frequencies on higher deuterium content (Fig. 1d). Indeed, the stretching vibration frequency of the H (D) in the C-C-H (C-C-D) complex depends on the reduce mass of the C-H (C-D) bonding. As the D mass is twice the mass of H atom, the vibration frequency of the C-D bonding is lower than the C-H one on increasing deuterium content.

### Isotopic effect on friction forces

**Figure 2:**
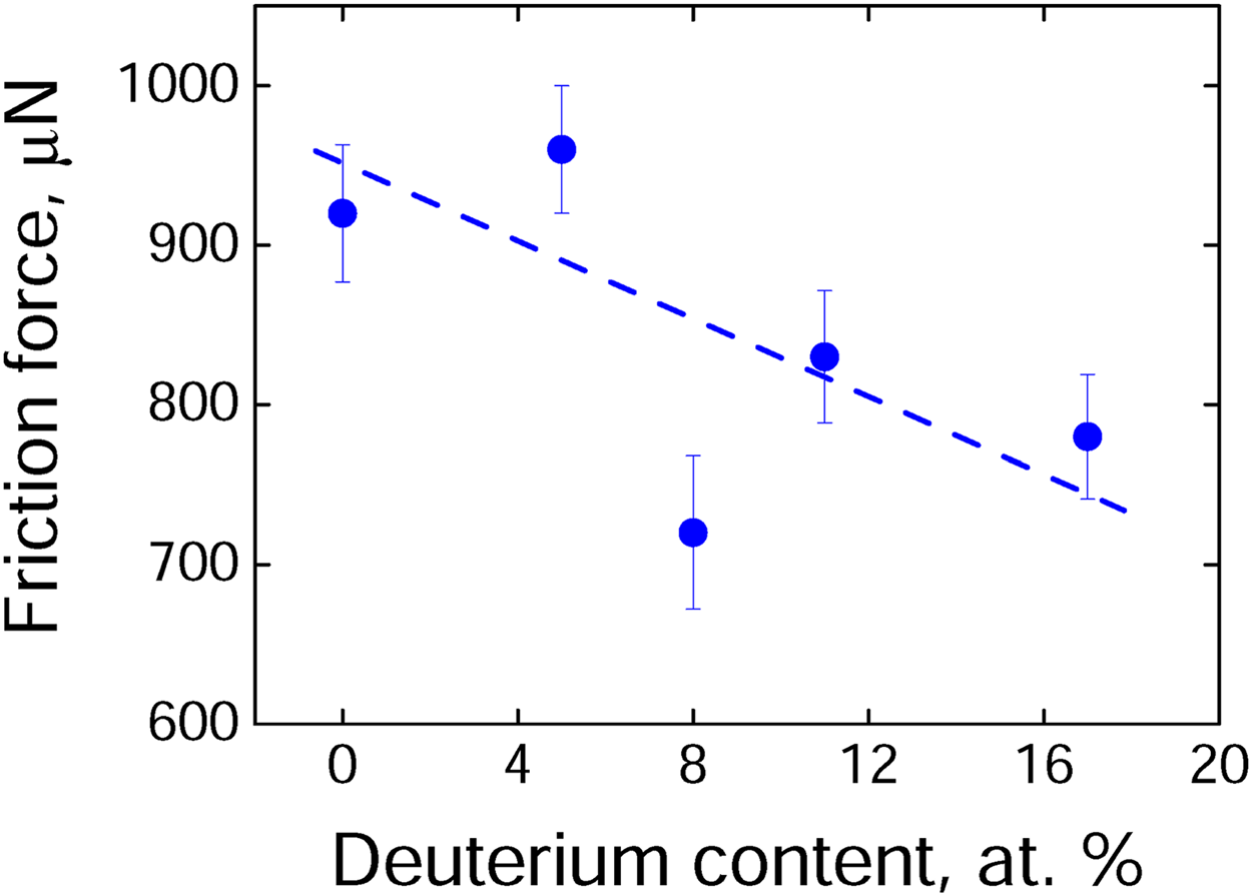
Friction force of a diamond spherical dome sliding on a-C:D/H thin films (isotopic systems) with different deuterium content at an average indentation depth of ~(75 ± 10) nm. Despite of the experimental dispersion data show on the plot, the decreasing tendency on deuterium content is observed.

Figure 2 shows the friction force acting on the tip sliding in relative motion and direct contact on flat a-C:D/H samples containing different amounts of deuterium (isotopic systems). Even though the experimental data dispersion, one can conclude that the increasing incorporation of deuterium relative to hydrogen in the a-C:D/H thin films diminishes the friction force of the nanotribological system. However, one must notice that both local minima and maxima points are quite interesting and these behaviors will be explained later on. Physically, the hydrogen substitution by deuterium in the amorphous carbon structure *does not change* the material electronic structure, i.e., the electronic properties of the material such as band structure and dielectric constants remain unalterable, leading to conclude that these possible effects are equivalent in the friction experimental findings. As commented above, the isotopic substitution only modifies the mass of the involved vibrating particle, i.e., the oscillator frequency **ω**_**D**_ **= (k/m**_**D**_ **)** ^**1/2**^ changes due to the heavier deuterium mass (**m**_**D**_) as compared with the one of hydrogen while stiffness of the oscillator (**k**) remains constant. Consequently, one must affirm that the different isotopic systems change only the phonon distribution. Moreover, as determined in a previous work^22^, the ERDA profiles guarantee a homogenous concentration of deuterium and hydrogen as a function of depth, which rules out any possible influence of the deuterium and hydrogen distributions on the friction results.

In order to identify the friction mechanisms involved in the studied isotopic systems we should discuss what are the main parameters accountable of the phenomenon. Table 1 shows the hardness, elastic modulus, plastic deformation index, elastic deformation index, and surface roughness of the samples. One must remark that neither hardness nor elastics modulus can be used, separately, in order to analysis plastic and elastic deformation mechanisms. Indeed, the H^3^/E^2^ ratio should be applied to analyze the resistance to plastic deformation and the H/E ratio to analyze the elastic strain to failure, respectively^25, 26^. Whereas, the higher the H^3^/E^2^ ratio (higher resistance to plastic deformation) the lower the energy dissipation, and the higher the H/E ratio (higher resistance to elastic strain) the higher the energy dissipation. Consequently, a detailed analysis of both ratios can determine the real contribution of mechanical properties in the coefficient of friction.

**Table 1:** Hardness (H), elastic modulus (E), plastic deformation index (H^3^/E^2^), elastic deformation index (H/E), and surface roughness (Rq) measured for the a-C:D/H thin films.

Sample	H (GPa)	E (GPa)	H^3^/E^2^ (GPa/plastic deformation index)	H/E (adimensional/elastic deformation index)	Rq (nm)
0% at. D/20% at. H	18 ± 2	100 ± 10	0.6 ± 0.1	0.18 ± 0.03	12 ± 3
5% at. D/14% at. H	18 ± 1	105 ± 5	0.53 ± 0.05	0.17 ± 0.01	13 ± 3
8% at. D/10% at. H	16 ± 2	95 ± 10	0.5 ± 0.1	0.17 ± 0.03	16 ± 2
11% at. D/7% at. H	16 ± 2	89 ± 8	0.5 ± 0.1	0.18 ± 0.03	12 ± 2
17% at. D/0% at. H	15 ± 2	92 ± 9	0.4 ± 0.1	0.16 ± 0.03	15 ± 3

**Figure 3:**
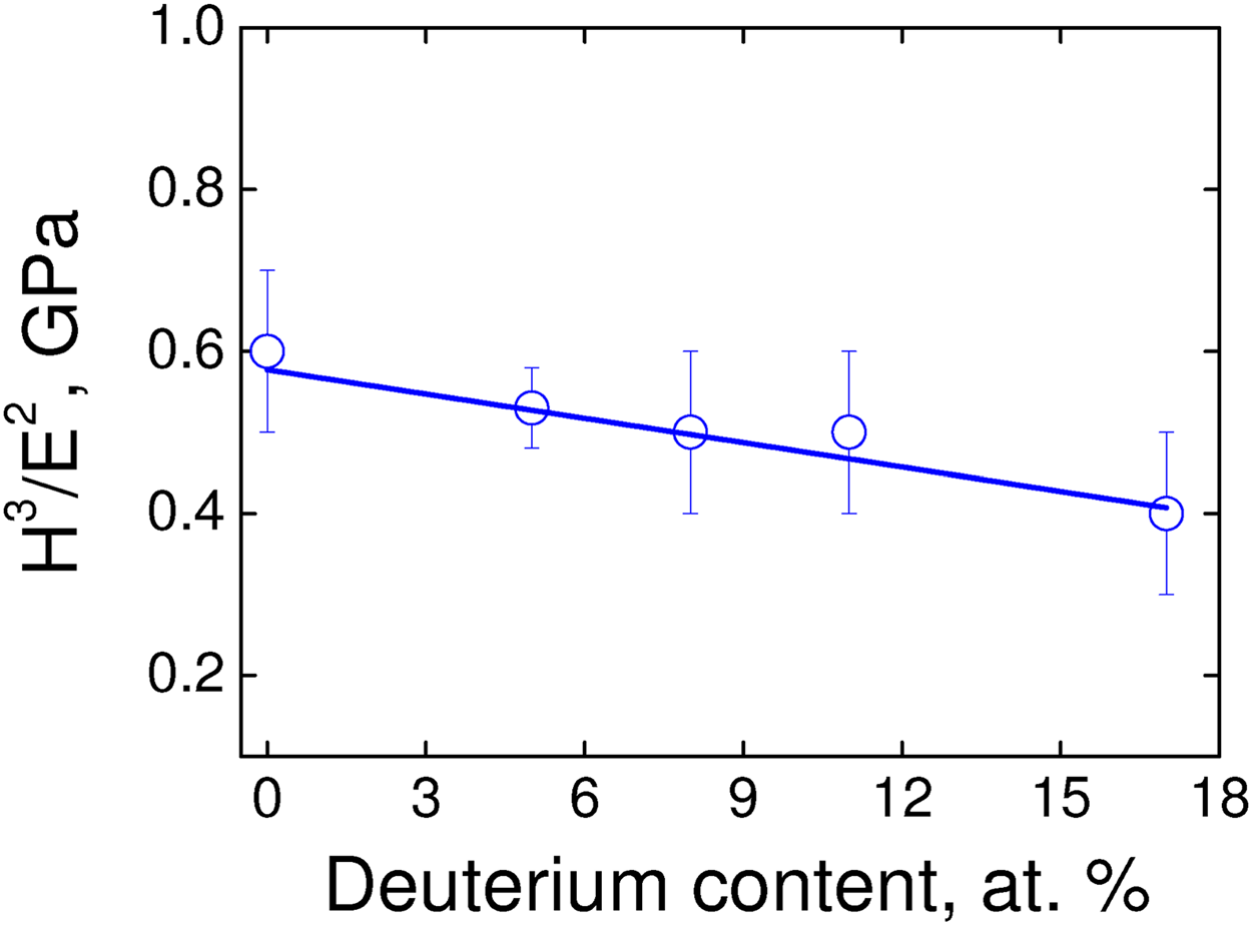
H^3^/E^2^ ratio as a function of the deuterium content in the a-C:D/H thin films (isotopic systems).

**Figure 4:**
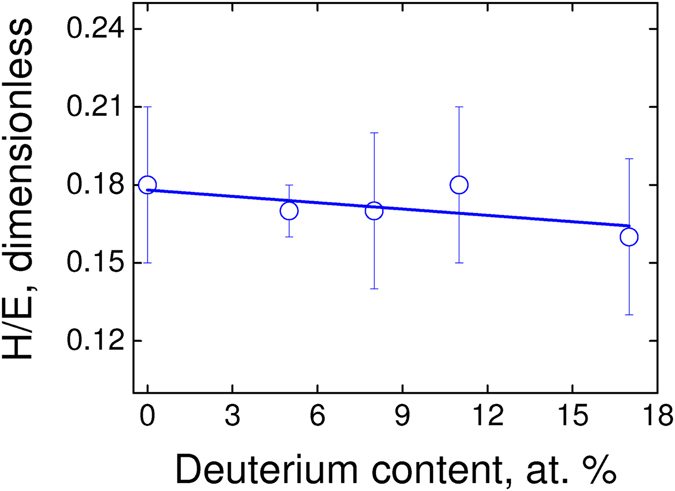
H/E ratio as a function of the deuterium content in the a-C:D/H thin films (isotopic systems).

As shown in Fig. 3, the H^3^/E^2^ ratio (plastic deformation index) diminishes up to 30%, which indicates that the highest dissipation energy must be expected for the sample with the highest deuterium content. Such an interpretation goes in the opposite way because we observed a lower coefficient of friction, i.e., a lower dissipation energy as higher the deuterium content. As shown in Fig. 4, the H/E ratio (elastic deformation index) diminishes up to 7%, amount representing a lower value than the experimental error (higher than 10%). Thus, the energy dissipation through an elastic deformation mechanism is roughly constant, independently of the deuterium content. According to these experimental evidences, the observed tendency in our results for an irreversible process of friction is not being conducted neither through plastic nor elastic deformation mechanisms as the main dissipation channel. Regarding the roughness, they are quite similar among samples ruling out interferences on the friction forces measurements. One must notice that the hardness slightly decreases with the increasing of the deuterium content (see Table 1) and consequently a higher tip contact area is expected. Therefore, a higher contact area *should increase* the friction forces, an effect that we did not observe in the experiments. Thus, a softer a-C:D thin film (higher contact area) cannot explain a lower friction. Furthermore, any wear such us trails and debris were detected after direct inspection of the sliding surface by FEG-SEM (not shown).

Finally, as it is well known, in the classical theory of mechanical contact friction studies, the hardness, elastic modulus, and roughness determine the actual (effective) contact area provided normal load indentation^5^. Therefore, one can conclude that different hardness and elastic modulus should imply different contact areas that will be calculated as contact area factors in order to evaluate the maximum and minimum points observed in Fig. 2.

### Classical contact mechanics to estimate the contact area

By using the Hertz’s theory of elastic bodies in non-adhesive contact mechanic, one can calculate, first, the radius (a) of the contact area as described in equations 1 and 2 ^27^:

1 $${\rm{a}}={(3{\rm{WR}}^{\prime} /{\rm E}^{\prime} )}^{1/3}$$

where W is the normal applied load, R′ is the reduced radius of curvature, and E′ the reduced elastic modulus.

2 $$1/{\rm{R}}^{\prime} =2/{{\rm{R}}}_{{\rm{A}}}$$

where R_A_ is the radius of sphere (in our case, the radius of the spherical dome of the conical tip). Finally, we can calculate the contact area as **π.a** ^**2**^.

**Figure 5:**
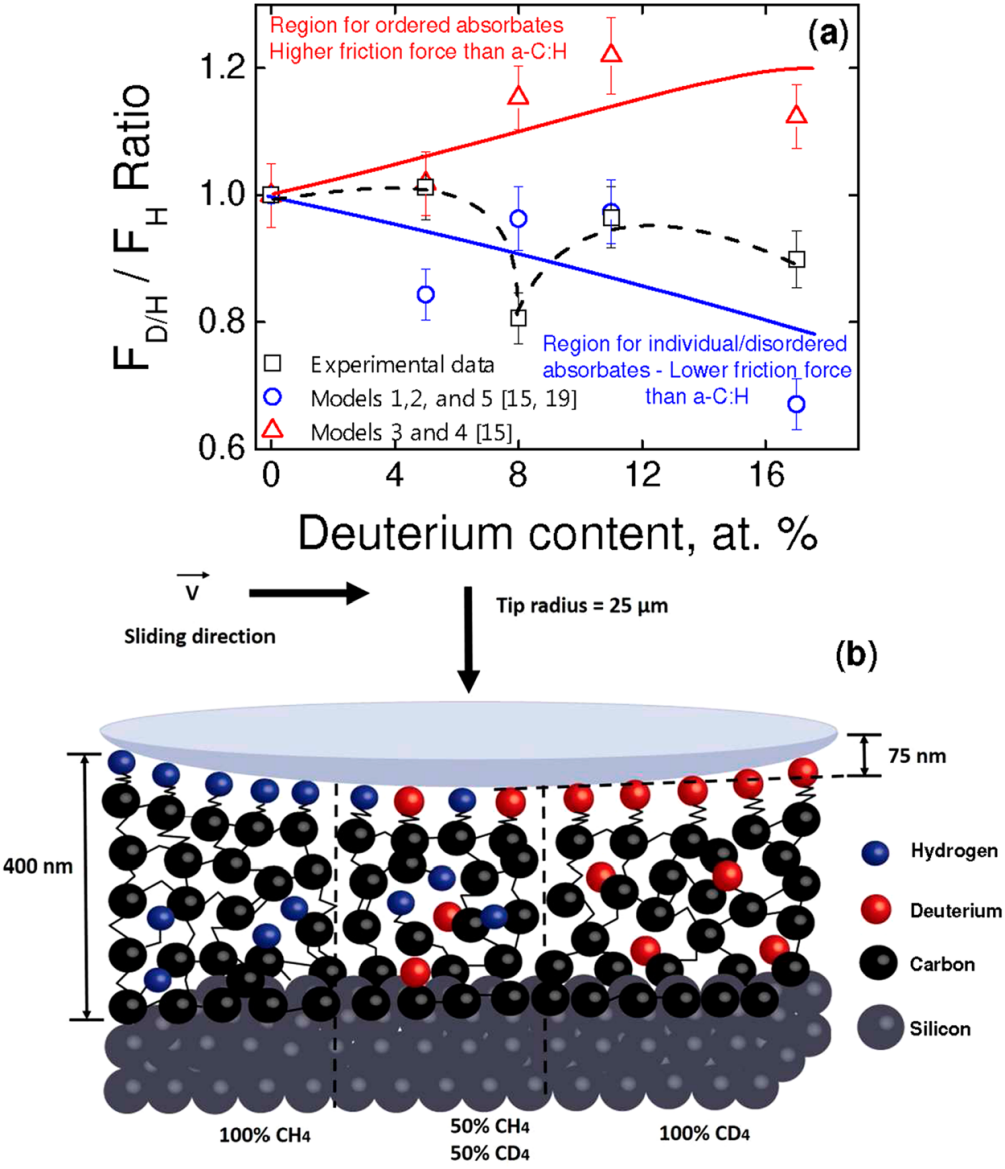
(**a**) F_H__/D_/F_H_ experimental ratio as a function of deuterium content (empty black squares). The empty red triangles correspond to the ordered commensurate adsorbate layer models for transverse (model #3) and longitudinal vibrations (model #4) and the empty blue circles correspond to the single adsorbate (model #1 and 2) and disordered adsorbate distribution (model #5) models after the calculations of F_H/D_/F_H_ ratio by using the parameters provided from these models. The solid lines represent the average tendency. The experimental data follow a dashed line Y-type (guide for the eyes) and fall in the region of models #1, 2, and 5. All the values were corrected by a contact area factor. (**b**) Schematic (not in scale) of the sliding of a diamond spherical dome on three different a-C:D/H thin films.

Table 2 summarizes the data used for the contact area and contact area factor calculations. The contact area factor is a term that corrects the different contact areas found in the experiments. The contact area factor will be applied in the following equation for the friction force due to vibrational damping and Fig. 5a in order to normalize the data.

**Table 2:** Reduced elastic modulus (E′), the contact area calculated by using the Hertz’s Theory (equations 1 and 2) and the contact area factor with a normal load (W) of 10 mN and a radius of the spherical dome of the diamond conical tip (RA) of 25 μm.

Sample	E′ (GPa)	Contact area, m^2^	Contact area factor (A of a-C:H/D/A of a-C:H)
0% at. D/20% at. H	119 ± 10	6.75E-12	1
5% at. D/14% at. H	125 ± 5	6.53E-12	0.97
8% at. D/10% at. H	113 ± 10	6.97E-12	1.03
11% at. D/7% at. H	107 ± 8	7.25E-12	1.07
17% at. D/0% at. H	109 ± 9	7.17E-12	1.06

### Isotopic effect on friction forces and phononic models

The phononic friction model for sliding surfaces takes into account energy dissipation by phonons coupling, i.e., the microscopic coupling interaction among surface oscillators leads to energy dissipation by non-conservative macroscopic forces associated with bulk phonon excitations. The friction force due to vibrational damping is giving by **F**_**f,vib**_ = **m**_**tip**_ **ηvσA** ^19^. Here, **m**_**tip**_ is the dynamical effective mass of the diamond tip, **η** is the damping constant of the inelastic interaction process, **v** is the sliding velocity between both surfaces in relative motion, **σ** is adsorbate areal density and **A** is the contact area. By knowing that the atomic densities of the a-C:D/H studied films are the same^22^, the above estimated contact area factors, and the dynamical effective mass of the diamond tip and sliding velocity are constant in all the measurements, the **η** is our most important parameter to analyze. From these considerations, one can conclude that the damping constant **η** is the key factor to compare in the experimental results, i.e., any observable change in the friction forces should be ascribed to the influence of deuterium in the energy dissipation process. Indeed, the deuterium content modifies only the phonon distribution where the last determines the damping constant (**η**).

As commented above, several models are invoked to explain the nano-scale phenomenon. Therefore, let us summarize five important models (from now on, model # 1 to 5) taking in account phonons damping process in sliding surfaces. In the case of damping processes involving parallel (||) or perpendicular (⊥) single adsorbate prompted vibrations via damping elastic waves, two different expressions for the damping constant have been proposed, namely **η**_**||(⊥)**_ **≅ mω**_**0**_^**4**^ **/8πρC**_**T**_^**3**^ **ξ**_**||**_ **(ξ**_**⊥**_ **)** (*model # 1*)^15^. Here **m** is the adsorbate’s mass, ***ω*** is the vibrational frequency, ***ρ*** is the density, ***C***_***T***_ is the transverse sound velocity, **ξ**_**||**_ (**ξ**_**⊥**_) are friction parameters that depend on the size of the adsorbate and in our case the value is ~3^15, 16^. A simplified scenario of this equation is giving by **η ≈ mω**_**0**_^**4**^ **/2πρC**_**T**_^**3**^ (*model # 2*)^19^. This simplification assumes that an elemental vibration associated with an adsorbate atom is though as coupling with an oscillators associated with the substrate resulting in a **ΔE** ≅ **E(m/m**_**s**_ **)** energy transference. Here **E** is the energy of the adsorbate elemental vibration and **m**_**s**_ is an “effective” mass of the substrate^19^. In the case of damping processes involving ordered commensurate adsorbate layers two different expressions for the dumping constant are obtained, namely **η**_**||**_ **= mω**_**0**_^**2**^ **n**_**a**_ **/ρC**_**T**_ (*model # 3*) and **η**_**⊥**_ **= mω**_**0**_^**2**^ **n**_**a**_ **/ρC**_**L**_ (*model # 4*)^15^. Here, ***n***_***a***_ is the number of adsorbates per unit area and ***C***_***L***_ is the longitudinal sound velocity. Physically, these expressions represent elastic waves propagating parallel (perpendicular) the material surface prompting a parallel (perpendicular) stress distribution. For completeness, finally, a model considering disordered commensurate adsorbate layers shows that the friction coefficient is giving by **η**_**0**_ **≅** **(1-θ) mω**_**0**_^**4**^ **/8πρC**_**T**_^**3**^ + **mω**_**0**_^**2**^ **n**_**a**_ **/ρ C**_**T**_ (*model # 5*)^15^ where the adsorbate are randomly distributed and **0** < **θ<1** represents the adsorbate coverage ratio. In these models, the adsorbate layer represents the surface responsible by the momentum interchange of the sliding parts and the bulk material energy dissipation, independently of the depth.

In order to compare the results obtained in the studied a-C:D/H materials with these models, the sound velocities (C_T_ and C_L_) are assumed to be given by the elasticity classical theory, i.e., **C**_**T**_ = **(E/ρ)** ^**1/2**^ ^28^. Moreover, it is generally assumed that **C**_**L**_ **~** **2C**_**T**_^15^. For the sake of simplicity, we shall consider the more probable and intense stretching vibrational frequency vibrational mode of the materials, i.e., 2120 cm^−1^ (C-D) and 2940 cm^−1^ (C-H). We remark that we have neglected the C-H and C-D scissoring, rocking, wagging, and twisting modes because we assume that there is not transference of momentum in those directions, which are mainly parallel to the sliding direction.

As we are focusing in the relative influence of the friction properties, it is worthy to plot the ratio of the friction dissipative forces, i.e., **F**_**D/H**_/**F**_**H**_. Figure 5a shows the F_D/H_/F_H_ ratio as a function of the deuterium content in the studied a-C:D/H thin films. For completeness, the theoretical results obtained by using the above-mentioned models are shown. In all cases, the results were corrected by the contact area factor that takes into account the different experimental contact areas previously calculated. After these considerations one can conclude that the experimental results fall in the tendency described by the single adsorbate vibration or disordered models (see black squares and blue circles in Fig. 5a). However, it is quite interesting to analyze the behavior of the experimental data after the contact area factor. While the maximum at 5 at. % (deuterium) in Fig. 2 roughly disappears, the minimum at 8 at. % (deuterium) remains. We believe that a new contribution should be incorporated into the phononic model, i.e., an entropic contribution. Thermodynamically, the entropy shows a maximum at 50%(D)–50%(H) (8 at. % (deuterium), which is expressed as a mixing entropy that increases the occupancy probability of disordered states.

Let us suggest, at the atomic scale, how the actual friction dissipative phenomenon is taking place (Fig. 5b). The interaction of the spherical dome diamond tip with the outermost layers of the a-C:D/H material dissipate energy via phonons, i.e., the *microscopic* intimated contact at nanoscale indentations with the unidirectional sliding is responsible of the *macro* scale dissipative friction phenomenon. Finally, it is remarked that both the *single* adsorbate and *disordered* distribution models fit fairly well the experimental results.

Summarizing, the following conclusions are drawn. First, the experimental setup implemented for the studied a-C:D/H material (isotopic system) allows to separate the phonon contribution from those such as electronic/electrostatic/magnetic contributions associated with friction dissipative forces. Second, the intimate contact obtained by dragging the nanoindentation tip on the a-C:D/H thin films shows that the more probable origin of the friction phenomenon stems from phononic dissipation mechanism. Indeed, the presence of deuterium in a-C:D/H decreases the macroscopic friction forces and the quantitative results are explained assuming *single* adsorbate and *disordered* distribution models compatible with the amorphous structure of the isotopic system. Finally, these findings may contribute to the understanding of phenomenological parameters like friction coefficient and shear stress intervening in the classical laws used in macroscopic phenomena.

## Methods

### Preparation of isotopic samples

Deuterated and hydrogenated amorphous carbon films (a-C:D/H) were deposited by plasma enhanced chemical vapor deposition (PECVD). Deuterated methane (CD_4_) and methane (CH_4_) mixtures were the precursor gases. The reactor background pressure was ~10^−4^ Pa. Oriented <100> silicon (substrates) were cleaned in an ultrasonic acetone bath (15 minutes) followed by 1 minute bath in distillate water and HF aqueous 10 vol. % solution. The samples were mounted on a water-cooled 7.5 cm-diameter copper cathode fed by a RF (13.56 MHz) power supply. The deposition pressure and incoming gas flux were 8 Pa and 10 sccm, respectively. The films thickness of ~400 nm is obtained in ~35 minutes of deposition. The self-bias voltage during deposition was fixed at 350 V and the CD_4_ partial pressure varied from 0% (a-C:H films) to 100% (a-C:D films).

### Physicochemical characterization of isotopic samples

Rutherford backscattering spectrometry (RBS) and elastic recoil detection analysis (ERDA) were used to determine the chemical composition of the a-C:D/H thin films. Details of these measurements are described elsewhere^22^. Structural details of the a-C:D/H thin films were studied by Raman spectroscopy (Raman Confocal NTegra Spectra NT-MDT/473 nm laser) and Fourier transform Infrared (FTIR) spectroscopy (PerkinElmer FTIR Spectrometer/model Spectrum 400).

### Nanotribological experiments in isotopic samples

The hardness and reduced elastic modulus of the a-C:D/H thin films were measured using a Hysitron TI 9000 Triboindenter with normal loads from 100 to 800 μN. The results were analyzed by the Oliver and Pharr method^29^. Nanotribological tests by direct contact at the nanoscale were performed by unidirectional scratching using a NanoTest-600 equipment (MicroMaterials Limited). The roughness and friction measurements were obtained by applying a normal load of 10 mN with a diamond *spherical* dome tip (25 μm radius). The samples were displaced at a rate of 1 μm.s^−1^ to a final scanning length of 680 μm. This velocity guarantees that the energy dissipation via the material phonon falls in an isothermal bath due to the 13 orders of magnitude between the vibration frequency (C-H and C-D) and the sliding velocity when the time scale is considered. Moreover, the extremely slow velocity of the tip displacement guarantees that not inertial forces are present in the experiment. After the sliding experiments, we analyzed the surfaces of the a-C:D/H thin films directly by FEG-SEM (Tescan MIRA3). In all cases, any wear (for example, trails or debris) was detected. The friction forces are obtained by averaging 20 (twenty) measurements performed for each sample. The average indentation depth was ~(75 ± 10) nm. The temperature and relative humidity for all hardness and friction measurements were (23 ± 1) °C and (50 ± 5) %, respectively.
